# Multidrug-Resistant* Bacteroides fragilis* Bacteremia in a US Resident: An Emerging Challenge

**DOI:** 10.1155/2016/3607125

**Published:** 2016-06-23

**Authors:** Cristian Merchan, Sunita Parajuli, Justin Siegfried, Marco R. Scipione, Yanina Dubrovskaya, Joseph Rahimian

**Affiliations:** ^1^Department of Pharmacy, NYU Langone Medical Center, New York, NY 10016, USA; ^2^Department of Infectious Diseases, NYU Langone Medical Center, New York, NY 10016, USA

## Abstract

We describe a case of* Bacteroides fragilis* bacteremia associated with paraspinal and psoas abscesses in the United States. Resistance to b-lactam/b-lactamase inhibitors, carbapenems, and metronidazole was encountered despite having a recent travel history to India as the only possible risk factor for multidrug resistance. Microbiological cure was achieved with linezolid, moxifloxacin, and cefoxitin.

## 1. Brief Case Report

An 82-year-old male with no significant past medical history was admitted to the intensive care unit for acute hypoxic respiratory failure, fever, hypotension, and confusion. Laboratory findings were significant for leukocytosis and elevated liver function tests. He was empirically started on vancomycin, cefepime, amikacin, and azithromycin for septic shock secondary to pneumonia. Within 24 hours of admission, 3 out of 6 blood cultures grew anaerobic Gram-negative rods, prompting a switch in antibiotics to piperacillin-tazobactam and metronidazole, pending susceptibility results. A computed tomography scan of the abdomen and a colonoscopy revealed no significant findings; however, the patient remained persistently febrile.

In the following two days, the patient's mental status improved and he began to report lower back pain. A magnetic resonance image (MRI) of the lower back was ordered and revealed discitis at the level of L2–L5 as well as paraspinal, epidural, and psoas abscesses.* Bacteroides fragilis* was identified from the blood cultures with resistance to ampicillin-sulbactam (MIC 32 mg/L), clindamycin (MIC 32 mg/L), metronidazole (MIC 32 mg/L), penicillin G (MIC > 32 mg/L), cefoxitin (MIC 12 mg/L), imipenem-cilastatin (MIC 1 mg/L), ertapenem (MIC 12 mg/L), and meropenem (MIC 32 mg/L). Further susceptibility testing demonstrated an increased MIC of 6 to imipenem, linezolid (MIC 2 mg/L), ceftazidime-avibactam (MIC 24 mg/L), ceftolozane-tazobactam (MIC 256 mg/L), and tigecycline (MIC 256 mg/L). All susceptibilities were confirmed by E test. High dose imipenem-cilastatin (1 gram intravenously every 6 hours) was initiated along with linezolid 600 milligrams intravenously every 12 hours. Given the high level of resistance, the patient was asked about any risk factors associated with multidrug-resistant pathogens. He reported having traveled throughout India four months prior to admission but was never hospitalized nor did he receive any antibiotics during his time abroad. In an attempt to obtain source control, general surgery was consulted and determined that the harm of a surgical intervention outweighed the benefits. Only a small amount of fluid was aspirated by Interventional Radiology and the culture was negative.

The bacteremia cleared after 3 days and the fevers resolved after 7 days. The patient was discharged after 2 weeks on the following antibiotics: cefoxitin, linezolid, and high dose imipenem-cilastatin. Linezolid was stopped a month later due to thrombocytopenia. Follow-up susceptibilities revealed that the* B. fragilis* was sensitive to moxifloxacin (MIC 0.125 mg/L) which was then added to the patient's regimen. The patient completed a total 12-week course of antibiotics and had normalization of his c-reactive protein and erythrocyte sedimentation rate. Follow-up MRI at 12 weeks showed significant reduction in the patient's paraspinal, epidural, and psoas abscesses.

## 2. Discussion

The* B. fragilis *group is the most common anaerobic organism recovered in blood cultures to date [10]. They are frequently isolated from the gastrointestinal tract but are rarely present in the oral cavity, upper respiratory tract, and female genitalia. Their slow in vitro growth, association with polymicrobial infections, and potential for antimicrobial resistance tend to complicate the treatment course [11].

The variation in susceptibilities for* B. fragilis* isolates depends on the individual species, country, medical institution, and antibiotic use within a geographic location [12]. The number of reports of multidrug-resistant* B. fragilis* strains has increased in the past decade as highlighted in Table 1 [1–9]. In particular, at NYU Langone Medical Center, resistance rates for 361 Bacteroides isolates were evaluated over a 5-year time period which demonstrated overall resistance rates of 5% (17/361) to metronidazole, 4% (13/361) to carbapenems, and 0.3% (1/361) to both carbapenems and metronidazole. Of note, 96% (16/17) of the isolates that were resistant to metronidazole were susceptible to carbapenems. Additionally, 94% (12/13) of the isolates that were resistant to carbapenems were susceptible to metronidazole. Our institution's higher resistance rates are in direct contrast to the rates reported in the USA for metronidazole (<1%) and carbapenems (1%) from 2008 to 2013 [12].

The mechanisms of antibiotic resistance in* B. fragilis* have been well described for carbapenems and metronidazole. Resistance to carbapenems is mainly mediated through the production of class b metallo-beta-lactamase enzymes encoded by the cfiA gene in the presence of an insertion sequence [12]. Metronidazole resistance has been associated with the presence of nitroimidazole resistance gene, nim A-G, that prevents the activation of metronidazole through the production of nitroimidazole reductase. The* B. fragilis* isolated from this patient revealed high-level resistance to multiple antibiotics including metronidazole, meropenem, and ertapenem. Resistance was also observed for ampicillin-sulbactam, clindamycin, and tigecycline. The only risk factor for a multidrug-resistant organism was a recent trip to India; however, he was not hospitalized or exposed to antibiotics as seen in other published reports. The* B. fragilis* isolates from our patient were analyzed by the Center for Disease Control and Prevention for Genomic Epidemiology database which identified that the isolates contained the following resistance genes: cfiA, cfxA, erm (F), erm (B), and sul2. From the analysis done using CLCbio and Geneious, it seems that there are no insertion sequences upstream of the gene; therefore the resistance to B-lactams seen phenotypically is most likely due to the cfxA gene. The cfx A gene leads to the production of a class a serine beta lactamase responsible for high-level resistance to cephalosporins [12]. In addition, the* B. fragilis *isolates did not contain nim genes conferring metronidazole resistance, suggesting that alternative resistance mechanisms like efflux pumps may be present [3].

In conclusion, this is the third case of MDR* B. fragilis* infection reported in a US hospital with resistance to both carbapenems and metronidazole; however, it is the first case in which the patient was not hospitalized abroad before returning to the USA [1, 2]. In addition, this is the first case that describes a monomicrobial* B. fragilis* bacteremia associated with a paraspinal, epidural, and psoas abscesses. Our case report suggests that physicians can no longer rely on the assumption of metronidazole or carbapenem susceptibility and should consider requesting susceptibility testing when treating severe infections caused by* B. fragilis.*

## Figures and Tables

**Table 1:** Previous case reports of *B.  fragilis *resistance and treatment regimens.

Case reports	Source of infection	Resistance mechanisms	Definitive treatment regimen	Outcome
*United States case reports*				

Sherwood et al. [1]	Bacteremia + wound	*nimE*	Moxifloxacin 400 mg IV q24 h + linezolid 600 mg IV q12 h Duration: 8 weeks	Survived

Kapalpila et al. [2]	Bacteremia + intra-abdominal fluid	No molecular investigation; resistant to MTZ^1^, imipenem, PTZ^1^, clindamycin, cefotetan, amp/sul^1^, moxifloxacin	Ertapenem 1 g IV q24 hr + Linezolid 600 mg IV q12 hr Duration: 4 weeks	Survived

*Non-United States case reports*				

Ank et al. [3]	Bacteremia	*cfiA, nimE, ermF, tetQ*	Moxifloxacin 400 mg IV q24 h + piperacillin-tazobactam 4.5 g IV q8 hr Duration: 7 days	Survived

Urbán et al. [4]	Abdominal fluid	*cfiA, nimA, erm, cepA, tetQ*	Locally applied antibiotic therapy/wound care	Survived

Hartmeyer et al. [5]	Bacteremia + intra-abdominal fluid	*cfiA, nimD, ermF, tetQ, tetX*	Meropenem + metronidazole Duration: 6 days, discontinuation due to death	Died

Katsandri et al. (2 case reports) [6]	Case 1: bacteremia from a colitis Case 2: esophagojejunal anastomotic leak	*cfiA*	Case 1: metronidazole 500 mg IV q8 h + cefotaxime 2 g IV q8 h Case 2: imipenem 500 mg IV q8 h	Case 1: died Case 2: died

Wareham et al. [7]	Bacteremia from pancreatitis	*cfiA, ermF, tetQ, efflux pump bmeB9/B15*	Linezolid 600 mg IV q12 h	Died

Rotimi et al. (3 case reports) [8]	Case 1: paracolic abscess Case 2: surgical wound Case 3: groin and scrotal abscess	No molecular investigation; resistant to MTZ	Case 1: imipenem 500 mg IV q8 h Case 2: amoxicillin-clavulanate acid 600 mg PO q8 h Case 3: meropenem 500 mg IV q8 h + cefepime + amikacin 500 mg IV q12 h	Case 1: survived Case 2: survived Case 3: died

Turner et al. [9]	Bacteremia from peritonitis and empyema	No molecular investigation; resistant to MTZ, imipenem, amoxicillin-clavulanate acid	Gentamicin and clindamycin	Survived

^1^MTZ: metronidazole; PTZ: piperacillin-tazobactam.
